# Supplementary material for: Rabies Vaccination of 6-Week-Old Puppies Born to Immunized Mothers: A Randomized Controlled Trial in a High-Mortality Population of Owned, Free-Roaming Dogs
Source: Trop Med Infect Dis. 2020 Mar 12;5(1):45. doi: 10.3390/tropicalmed5010045 (PMC7157201; doi:10.3390/tropicalmed5010045)
Supplement: Supplementary file 1 [file tropicalmed-05-00045-s001.pdf]

Tropical Medicine and Infectious Disease

MDPI

Article

Rabies Vaccination of 6-Week-Old Puppies Born to Immunized Mothers: A Randomized Controlled Trial in a High-Mortality Population of Owned, Free-Roaming Dogs

Sintayehu Arega [1], Anne Conan [1], Claude T. Sabeta [2,3], Jan E. Crafford [3], Jeanette Wentzel [4], Bjorn Reininghaus [5], Louise Biggs [6], Andrew L. Leisewitz [7], Melvyn Quan [3], Felix Toka [8] and Darryn L. Knobel [1,3,*]

[1] Center for Conservation Medicine and Ecosystem Health, Ross University School of Veterinary Medicine, Basseterre, St Kitts and Nevis; SintayehuArega@students.rossu.edu (S.A.); AConan@rossvet.edu.kn (A.C.);
[2] Agricultural Research Council-Onderstepoort Veterinary Institute, OIE Rabies Reference Laboratory, Onderstepoort 0110, South Africa; SabetaC@arc.agric.za
[3] Department of Veterinary Tropical Diseases, Faculty of Veterinary Science, University of Pretoria, Onderstepoort 0110, South Africa; jannie.crafford@up.ac.za (J.E.C.); melvyn.quan@up.ac.za (M.Q.)
[4] Hans Hoheisein Wildlife Research Station, Faculty of Veterinary Science, University of Pretoria, Orpen 1364, South Africa; jeanette.wentzel@up.ac.za
[5] Mpumalanga Veterinary Services, Department of Agriculture, Rural Development, Land and Environmental Affairs, Thulamahashe 1365, South Africa; bjorn.reininghaus@gmail.com
[6] Department of Production Animal Studies, University of Pretoria, Onderstepoort 0110, South Africa; louise.biggs@up.ac.za
[7] Department of Companion Animal Clinical Studies, University of Pretoria, Onderstepoort 0110, South Africa; andrew.leisewitz@up.ac.za
[8] Center for Integrative Mammalian Research, Ross University School of Veterinary Medicine, Basseterre, St Kitts and Nevis; FToka@rossvet.edu.kn
* Correspondence: dknobel@rossvet.edu.kn

Table S1. Results of sensitivity analysis for survival analysis (six through 13 weeks of age), considering subjects reported as lost or stolen by owners as dead (n = 22).

	Sterile Water		Rabies Vaccine		HRs (95% CI) for RV within Strata of Sex
	Mortality Rate [a] (Deaths/Dog-Years)	HR (95% CI) [b]	Mortality Rate [a] (Deaths/Dog-Years)	HR (95% CI) [b]	
Females	1,808 (15/8.3)	1 (reference)	3750 (32/8.5)	1.71 (0.87–3.34)	1.71 (0.87–3.34)
Males	3,496 (30/8.6)	1.62 (0.80–3.29)	2744 (25/9.1)	1.36 (0.68–2.71)	0.84 (0.46–1.55)

Notes: HR = hazard ratio. CI = confidence intervals; [a] Mortality rate per 1,000 dog-years; [b] Compared to reference category (control females).

Table S2. Results of sensitivity analysis for survival analysis (six through 13 weeks of age), censoring subjects that reportedly died from accidents (n = 5).

	Sterile Water		Rabies Vaccine		HRs (95% CI) for RV within Strata of Sex
	Mortality Rate [a] (Deaths/Dog-Years)	HR (95% CI) [b]	Mortality Rate [a] (Deaths/Dog-Years)	HR (95% CI) [b]	
Females	844 (7/8.3)	1 (reference)	2696 (23/8.5)	2.83 (1.12—7.15)	2.83 (1.12–7.15)

Males	2914 (25/8.6)	3.37 (1.29–8.79)	2,195 (20/9.1)	2.23 (0.89–5.60)	0.66 (0.33–.34)

Notes: HR = hazard ratio. CI = confidence intervals; [a] Mortality rate per 1,000 dog-years; [b] Compared to reference category (control females).
